# Superhydrophilic Nanotextured Surfaces for Dental Implants: Influence of Early Saliva Contamination and Wet Storage

**DOI:** 10.3390/nano12152603

**Published:** 2022-07-28

**Authors:** Marcel F. Kunrath, André Correia, Eduardo R. Teixeira, Roberto Hubler, Christer Dahlin

**Affiliations:** 1 Department of Biomaterials, Institute of Clinical Sciences, The Sahlgrenska Academy at University of Gothenburg, P.O. Box 412, SE 405 30 Göteborg, Sweden; 2 Department of Dentistry, School of Health and Life Sciences, Pontifical Catholic University of Rio Grande do Sul (PUCRS), Porto Alegre 90619-900, Brazil; eduardo.teixeira@pucrs.br; 3 Materials and Nanoscience Laboratory, Pontifical Catholic University of Rio Grande do Sul (PUCRS), Porto Alegre 90619-900, Brazil; hubler@pucrs.br; 4 Universidade Católica Portuguesa, Faculty of Dental Medicine, Centre for Interdisciplinary Research in Health, 3504-505 Viseu, Portugal; andrecorreia@ucp.pt

**Keywords:** anodized surfaces, hydrophilicity, saliva, nanotopography, TiO_2_ nanotubes, dental materials, biomedical implants

## Abstract

Hydrophilic and nanotextured surfaces for dental implants have been reported as relevant properties for early osseointegration. However, these surface characteristics are quite sensitive to oral interactions. Therefore, this pilot study aimed to investigate the superficial alterations caused on hydrophilic nanotubular surfaces after early human saliva interaction. Titanium disks were treated using an anodization protocol followed by reactive plasma application in order to achieve nanotopography and hydrophilicity, additionally; surfaces were stored in normal atmospheric oxygen or wet conditioning. Following, samples were interacted with saliva for 10 min and analyzed regarding physical–chemical properties and cellular viability. Saliva interaction did not show any significant influence on morphological characteristics, roughness measurements and chemical composition; however, hydrophilicity was statistically altered compromising this feature when the samples were stored in common air. Cellular viability tested with pre-osteoblasts cell line (MC3T3-E1) reduced significantly at 48 h on the samples without wet storage after saliva contamination. The applied wet-storage methodology appears to be effective in maintaining properties such as hydrophilicity during saliva interaction. In conclusion, saliva contamination might impair important properties of hydrophilic nanotubular surfaces when not stored in wet conditions, suggesting the need of saliva-controlled sites for oral application of hydrophilic surfaces and/or the use of modified-package methods associated with their wet storage.

## 1. Introduction

The application of nanotechnology on biomedical implant surfaces has been broadly investigated in the last years due to the possibility of nano-scale interaction with local cells [1,2,3]. Methodologies aiming at nanostructuration and nanomorphology on implant surfaces have been exploring improvements on the healing process and infection control [1,2,3,4]. Nano-scale surfaces demonstrated accelerated bone cell adhesion and increased parameters for osseointegration in vivo [1,2,3,4,5]. Additionally, specific nanostructures showed potential to reduce the adhesion and colonization of classic bacteria detected in biomedical implant contaminations [1,2,3,4,5,6].

Anodization is an electrochemical process that promotes the development of TiO_2_ nanotubes when applied on Titanium (Ti) surfaces with specific conditions [7,8]. The creation of nanotubes on dental implant surfaces showed promising features for better implant healing, such as quick cell-biomaterial interaction, antibacterial properties, improvement of osseointegration properties, hydrophilic characteristics and potential for the development of drug-delivery systems [1,4,9,10]. However, commercial dental implants using nanotubes and/or nanoporous surfaces are still scarce in the market, and some concepts about how these surfaces might react in the oral environment remain unclear. Meanwhile, saliva is a biological fluid present in all humans, and frequently is the first solution to interact with a biomaterial placed in the oral cavity [11]. Saliva is almost exclusively composed by water (99%); however, it is well known that different substances such as proteins, cells, bacteria and minerals are also present in its composition [11,12]. Therefore, saliva interaction with nanotubular surfaces might promote a thin organic pellicle formation and thus alter some surface properties designed to arrive intact at the surgical site.

The formation of a salivary pellicle over different implant surface topographies revealed changes in the surface wettability conditions [13,14] and reduction in cellular viability [15], and some studies also reported that this saliva layer might promote early bacterial colonization [16,17]. Implant surfaces with hydrophilic characteristics aim for a quicker adhesion and faster spread of bone cells when placed intra-osseous [18]. Nanotubular surfaces with super-hydrophilic characteristics showed increased bone-cells activity and significant expression of important bone formation-related genes when compared to hydrophobic surfaces [10,19]. Moreover, clinical studies reported that hydrophilic implant surfaces possess properties that might allow early loading of dental implants compared to other surface treatments [20]. Thus, it might be hypothesized that this exclusive surface property requires to be free of any impairment when interacting with the bone site to promote its complete beneficial responses. For that reason, companies and current studies have proposed the wet storage of dental implant surfaces until their surgical application in order to prevent impairment of their hydrophilic features [10,21,22,23,24,25]. Additionally, saliva contamination at surgical sites confectioned for implant insertion constitutes a complex issue due to the difficulty to control your invasion to the site [26]. Clinical situations involving stent-guided surgeries [27], patients with hypersalivation [28], patients with special mental disabilities [29], surgeon inattention, among other factors, may generate the early saliva interaction prior to the interaction with bone tissues.

Therefore, the aim of the present pilot study was to investigate the impact of early saliva interaction on the properties of anodized hydrophilic surfaces with or without wet storage, this study promoted their early contact with human saliva, identifying first their chemical–physical properties before saliva interaction and compared their possible alterations after saliva exposure. In addition, basic cell viability assays were performed to demonstrate biological differences caused by early saliva pellicle formation in vitro with pre-osteoblast cell lines.

## 2. Materials and Methods

### 2.1. Surface Treatment

A titanium (Ti) grade II plate (TitanioBrasil, São Paulo, Brazil) was used to manufacture 70 Ti disks (1 mm thick and 6 mm wide). The samples were cleaned with a 70% ethanol solution (Sigma, St. Louis, MO, USA,) and manually polished using emery papers (SiC papers—grit sizes 300, 600 and 900) to achieve a regularly polished surface. The anodization process followed a previously described methodology [10], in which the disks were double-attacked with acid solutions (hydrochloric acid, HCl—38% and sulfuric acid, H_2_SO_4_) during 1 h in order to create a rougher surface. The samples were washed and dried, then placed in an ultrasonic bath (25 kHz) with an electrolytic solution composed of ethyleneglycol, 0.5% ammonium fluoride (NH_4_F), 10% deionized (DI) water at a controlled voltage of 40 V for 1 h. The Ti samples were used as the anode and a Pt (platinum) sheet was used as the cathode. The solution temperature and bath were kept at 10 °C during the entire process. After anodizing, the samples were cleaned with DI water, isopropylic ethanol, acetone and DI water again, finishing with drying in N_2_.

**Figure 1:**
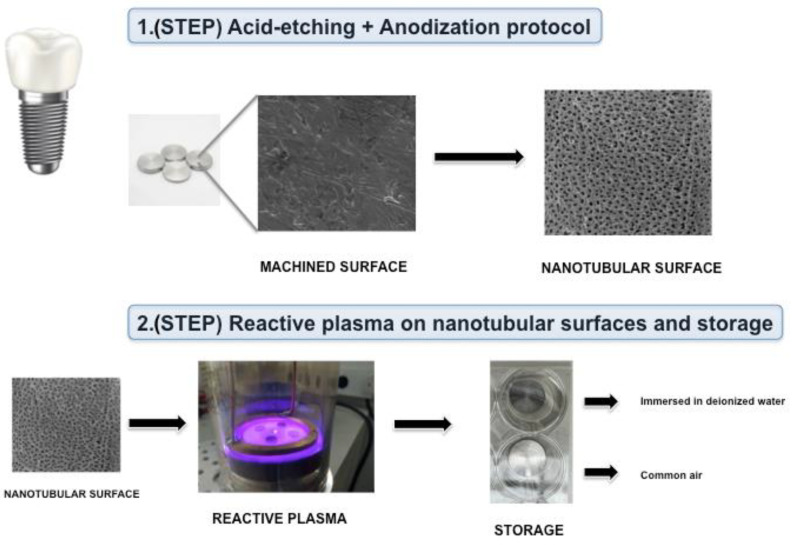
Scheme showing the methodological steps to achieve the hydrophilic nanotubular surfaces and methods for storage of disks. 1.(STEP)—Titanium disks with machined surfaces were treated using an anodization process in order to achieve nanotopography. 2.(STEP)—Nanotubular surfaces were submitted to reactive plasma application and directly stored in sterilized culture plates with DI water or common air.

In order to active hydrophilicity, a reactive plasma treatment was performed as previously described [10]. In summary, a vacuum chamber was used to apply reactive plasma of Ar/O_2_ for 5 min with a controlled power of 30 W over the anodized surface, promoting hydrophilic characteristics. Furthermore, the anodized hydrophilic samples were stored with two different protocols (wet storage and common air storage) using sterilized cell-culture plates with or without DI water added to the wells, in order to investigate the outcomes after saliva interaction with different storage protocols (Figure 1).

### 2.2. Surface Characterization

To evaluate topographical characteristics achieved by surface treatments prior to and after saliva interaction, a scanning electron microscope (FESEM, Inspect F50, Prague, Czech Republic) coupled with energy dispersive X-ray spectrometry system (EDS, Oxford, UK) and a transmission electron microscope (TEM, Tecnai G2 T20, Prague, Czech Republic) were applied (*n* = 3 per surface treatment). For wettability properties assessment prior to and after saliva interaction, the sessile drop method using a Goniometer—Contact Angle Measure (Phoenix 300, SEO, Kosekdong, Korea) equipped with DI water was applied. The samples (*n* = 5 per surface treatment) were exposed to a droplet of 0.012 mL and analyzed using a specific software (Surfaceware8, version 10.11, Kosekdong, Korea).

In order to investigate roughness parameters prior to and after saliva interaction, an atomic force microscope (AFM, Dimension Icon, Bruker, Billerica, MA, USA) was used. Three analyses were made in triplicate (*n* = 6 per group) at three different sites (machined discs, anodized discs and saliva contaminated discs (donor 1 and 2), with the cut-off value of 30 μm [30].

### 2.3. Saliva Collection and Interaction

Saliva samples were collected from two different healthy volunteers with absence of oral diseases and different ages (33 years old male and 62 years old female). An informed consent document was made and signed by the donors in order to consent to saliva sample collection and analysis. This study was approved by the Ethics committee of Pontificia Universidade Católica do Rio Grande do Sul (no. 7467). Prior to the contamination of the anodized surfaces, a collection of unstimulated whole saliva (WS) was performed in order to maintain similar conditions to the natural saliva composition. The donors performed mouth hygiene early in the morning and the saliva collection was completed before lunchtime, in order to simulate a realistic clinical situation. Sublingual saliva was collected using two cotton rolls placed under the volunteer’s tongue for 2 min. The cotton rolls were transferred to a 15-mL sterile tube with a 1-mL pipette tip for saliva collection after centrifugation at 10,000× *g* for 10 min [31]. Then, machined and anodized samples (wet storage and air storage)—see Table 1 for groups description—were exposed to the saliva for 10 min in sterile tubes and removed immediately for physical–chemical characterization and biological assays.

### 2.4. Biological Assays

For the biological assays, samples were sterilized previous to saliva contamination using UV-light for 30 min. After that, the saliva interaction was performed only on the anodized surfaces for 10 min, aiming to demonstrate the possible alterations caused by saliva in this specific surface treatment.

The evaluation of cell viability between the different surfaces, meaning non-treated surface (control), anodized surface and anodized saliva-contaminated surface (test, with saliva from donors 1 and 2) was performed utilizing murine preosteoblast cell line MC3T3-E1 (Sigma Aldrich, St. Louis, MO, USA). Cells were cultivated using Dulbecco’s modified Eagle’s medium/low glucose (DMEM, Gibco BRL, Gaithersburg, MD, USA) with 3.7 g L^−1^ sodium bicarbonate, 2.5 g L^−1^ HEPES, 10% fetal bovine serum (Cultilab, São Paulo, Brazil) and 1% penicillin/streptomycin (Gibco). After reaching 80% confluence, cells were detached from culture plates by pronase incubation. Three samples of each group were used for the assays and the test repeated three times. The cells (initial density 1 × 10^4^ cells/cm^2^) were seeded on each different surface group and evaluated by the 3-(4,5-dimethylthiazol-2-yl)-2,5-diphenyl tetra-zolium bromide assay (MTT, Sigma-Aldrich, St. Louis, MO, USA) at 24 h and 48 h time-points. Briefly, after exposure, the disks were washed with PBS and placed into a new 96-well plate. Then, the new solution of α-MEM (400 μL) and MTT (100 μL) was added. The solution was then cleared and the plate was put in a constant temperature incubator at 37 °C for 4 h. After that, 300 μL of dimethyl sulfoxide (DMSO) was added. Lastly, 200 μL of DMSO solution was transferred to a 96-well plate [10]. For solution vehicle control, DMSO was applied in order to achieve similar comparisons to the other groups. The absorbance was measured with a spectrophotometric microplate reader (Bio-Rad 600, San Diego, CA, USA) at a wavelength of 490 nm to assess cell viability [32].

### 2.5. Statistical Analysis

Data were demonstrated as means ± standard deviation (SD). For continuous data (roughness, viability and wettability), comparisons between groups were made applying the Student’s *t*-test. One-way ANOVA followed by post hoc testing (Tukey HSD), was used in further comparisons. Characterization analyses and viability assays were performed in triplicate. When roughness was considered, the NanoScopeAnalysis^®^ 1.40 software, Bruker, Billerica, MA, USA, was applied following a specific set of parameters [33]. Prism 9 (GraphPad software, San Diego, CA, USA) was applied in statistical analyses and significant differences were characterized at 5% (*p* < 0.05).

## 3. Results and Discussion

### 3.1. Surface Morphology

Surface treatments applying anodization as the main process for the development of nanotopographies have been widely used and reported in the last years [1,4,34]. However, there are several methodologies reported that have employed distinct steps resulting in a high number of different TiO_2_ nanotube morphologies. The process of anodization may be significantly altered by modifications in components and parameters applied in the electrochemical process such as the electrochemical solution, time of treatment, applied voltage, system temperature, sample pre-treatments and post-treatments and also the applied aging electrolyte [7,8,35,36]. Therefore, the development of oriented or disoriented nanotubes alignment [37], modification of nanotube dimensions (inner size and diameter) [38], alterations in their crystalline phase [10,39], and changes in the nanotube mechanical resistance [40] may be modified with specific intentions.

**Figure 2:**
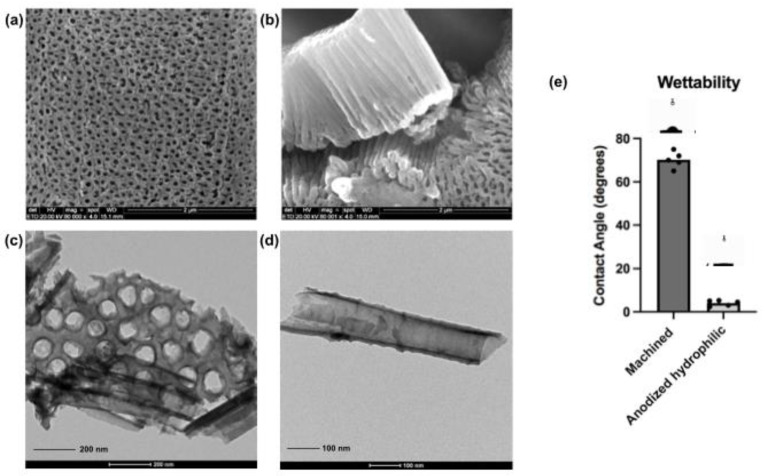
Morphological characterization and hydrophilicity properties of the nanotubular surfaces. Scanning electron microscopies from the anodized surfaces after surface treatment protocol, perpendicular view (**a**) and lateral view of the nanotubes (**b**) (SEM magnification—80,000×, scale bars—2 μm); Transmission electron microscopies from the anodized surfaces showing the nanotubular entrances and complete TiO_2_ nanotubes structure (**c**,**d**) (TEM scale bar—200 nm and 100 nm respectively). Additionally, contact angle measurements comparing control surfaces (machined) and anodized surfaces after reactive plasma application (**e**). The plasma application activated superhydrophilicity on the nanotubular surfaces.

The surface treatment applied in the present investigation developed a uniform and consistent nanotubular layer over the machined Ti samples, as verified in the microscopy images (Figure 2a–d). The transversal section image confirmed the complete formation of TiO_2_ nanotubes with an approximate length of 852 ± 56 nm from a lateral view (Figure 2b). Additionally, transmission electron microscopies (Figure 2c,d) showed the entire integrity of the nanotube walls throughout their entire structure, with entrance holes measuring 68 ± 5 nm and an intertubular space of 10 ± 4 nm. Current reports have demonstrated better osteogenic cells responses when in contact with nanotubular or nanoporous surfaces presenting entrance diameters around 70 nm compared to other dimensions [10,41]. Moreover, this specific nanotube size has been said to promote higher cell adhesion and higher expression of associated genetic bone formation markers [41,42].

After the anodization process, samples were exposed to the reactive plasma in a dedicated vacuum chamber aiming higher surface hydrophilicity. Implant surfaces with superhydrophilic characteristics have demonstrated a positive early response when associated with bone-related cells [10,18,20]. Additionally, in vivo and clinical studies showed that hydrophilic surfaces revealed higher osseointegration parameters compared to standard surfaces [43,44]. The measured contact angle by a goniometer evidenced the obtained difference in terms of hydrophilicity, from 70° ± 4° (machined surface) to 5° ± 2° (anodized surface with plasma), demonstrating the activation of high-hydrophilic surface features. In addition, characterizations of crystalline phase, surface morphology, wettability and chemical composition before and after the reactive plasma application were previously reported by our group [10]. The wet storage methodology applied here after activation of superhydrophilicity could be justified by the generated protection of surface properties until their application in the experiments. Choi et al. [45] demonstrated that the wet storage of Titanium and Zirconia disks could prevent the oxidation of the superficial layer of the analyzed materials, and also block the surface contamination by decreasing the level of Carbon that may induce changes in surface wettability. Furthermore, the wet storage proved to stabilize the hydrophilicity characteristics with successful results due to the surface hydration [10,22,23,24,45,46]. In the last two decades, companies and researchers have shown the significance of wet storage after surface treatments in commercial dental implants in order to maintain hydrophilic conditions and improve early biological outcomes [22,23,24,25].

### 3.2. Surface Properties before/after Saliva Interaction

Saliva is always present in the oral environment (gingiva, teeth, bone, blood) before any surgical procedure starts. The complexity of saliva interaction control during the placement of oral biomaterials such as dental implants or materials for bone regeneration is challenging, being more demanding in procedures involving more than one professional handling the case [26].

In order to induce the possible differences in surface characteristics created by the early saliva interaction (pellicle formation), the anodized hydrophilic surfaces were embedded for 10 min in natural human saliva and submitted to surface tests before and after the saliva contamination. The time of 10 min was chosen in order to simulate an eventual early interaction of the implant surface with saliva in an implant insertion procedure.

In this specific study, the saliva gathering was made using cotton rolls and centrifugation for a faster collection [31]. However, the use of cotton rolls may generate some influence in the total number of cells, bacteria and adsorbed proteins, and should be considered when compared with different studies. Several authors reported different methodologies to collect the saliva and apply it in their studies, even associated with techniques of freezing, filtration or direct application from the subject [14,31,47,48]. The application of saliva immediately removed from humans showed to be the most similar condition to the clinical environment [26]; however, it is extremely difficult to perform an entire study with human subjects providing immediate saliva excretion in laboratories without a proper process of storage. Therefore, this investigation was performed on different days, using one day for each surface analysis with a direct saliva collection. Moreover, as a limitation regarding the used saliva samples, this study selected only two different donors with extremely good oral health, introducing saliva with characteristics that might possibly generate different outcomes if compared with saliva samples derived from donors with poor oral conditions or oral diseases.

#### 3.2.1. Roughness Parameters and Superficial Changes

Superficial roughness measured around 1.5 μm have been considered by several authors as the best topographical condition for bone cell adhesion and successful osseointegration, due to an improved connection between the substrate and the cellular membrane when compared to smooth or highly-rougher surfaces [49,50]. The anodized surface proposed here achieved a roughness around 1.37 μm (Sa roughness parameter), corroborating to be quite similar to the gold standard for bone cells [49,50]. This specific roughness was achieved by the combination of firstly an acid-attack treatment aiming to add roughness to the smooth Ti surface, followed by an anodization process aiming to create a nanotopography. The salivary pellicle formation seemed to reduce in nano-scale metrics some of the measured roughness parameters, although no statistically significant differences could be observed between the anodized groups, as shown in Table 2. This could be observed probably due to the deposition of a thin saliva layer over the nanotubular surface, covering some of the depressions created by the surface treatment. The creation of this thin salivary layer was said to be a possible factor to induce changes in the chemical bonds between substrate and the substance deposited on the surface [16,51]. Moreover, the actual impact of this pellicle between the implant surface and tissue cells has not been clearly explained in the literature, but might be justified as it alters important surface properties such as roughness and wettability.

**Figure 3:**
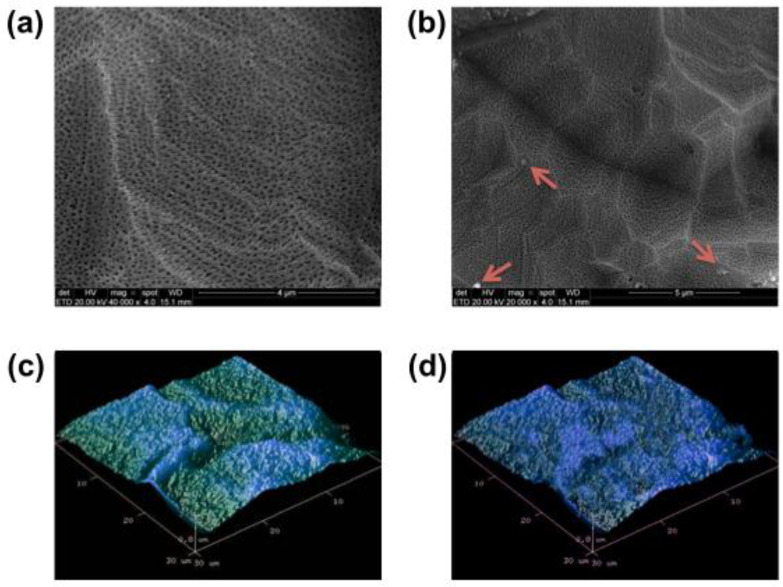
Superficial morphology acquired by SEM and by AFM of nanotubular surfaces before ((**a**)—SEM, scale bar—4 μm and (**c**)—AFM) and after ((**b**)—SEM, scale bar—5 μm and (**d**)—AFM) saliva interaction for 10 min. Small impurities (red arrows) can be observed after saliva exposition (**c**); however, no significant morphological alteration on the nanotubular surface could be detected.

Superficial morphological alterations were not found by electron microscopy. The surface morphology remained intact after saliva interaction, as seen in Figure 3. However, the application of EDS in specific surface areas after saliva interaction revealed some different chemical elements (impurities) in the spectrum (Table 3). The detection of oxygen reduced substantially, demonstrating that the reactivity promoted by free oxygen radicals on the anodized hydrophilic surfaces has been modified. Moreover, the chemical composition of human saliva may vary significantly between subjects in terms of saliva proteins, cells, minerals and bacteria, so some differences in terms of present components should be expected when comparing contaminated with intact surfaces [14,15].

#### 3.2.2. Wettability

**Figure 4:**
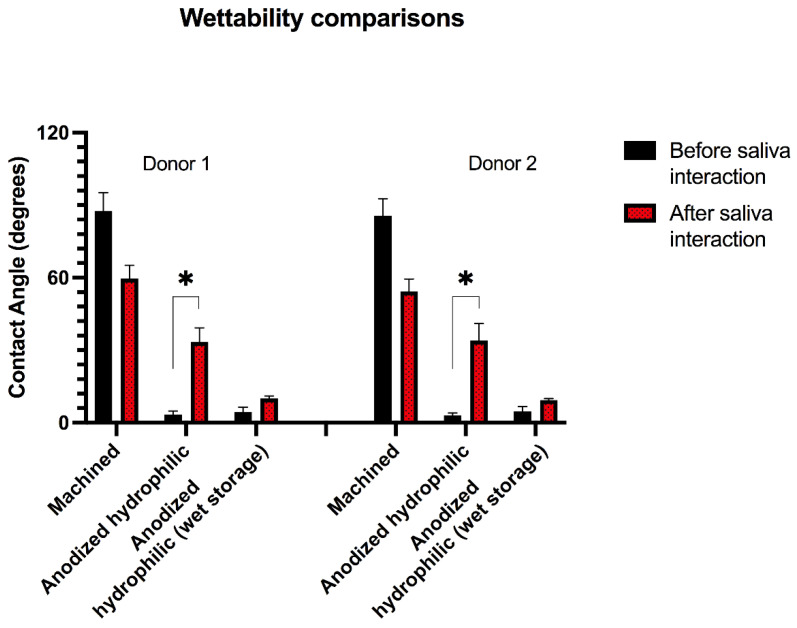
Wettability analysis before and after saliva interaction of two saliva donors. * Asterisks indicate statistical significance (*p* < 0.05) after saliva interaction for 10 min among the tested surface groups.

Hydrophilicity is a surface property that has been considered as primordial for quick osseointegration and fast bone healing of Ti implants [52]. Hydrophilic surfaces have been said to promote an early impact in the blood proteins and in the first cells to interact with the implant surface [53]. The acceleration in the healing process is provided by a better cell spreading around the implant surface associated with improved cell metabolism [52,53,54]. As discussed previously, the surface treatment applied in the present investigation developed a surface with high-hydrophilicity, aiming to promote a better local cell response when applied in in vivo environments. However, after the saliva interaction, significant changes occurred in the surface hydrophilicity measurements, as shown in Figure 4. The wettability characteristics changed significantly on the group with saliva interaction and without wet storage, increasing the contact angle in the wettability assessment. This might be explained by the formation of a salivary pellicle over the Ti surface, changing its high reactive property created by the proposed surface treatment, modifying it into a novel physical–chemical layer that reveals a new interaction between contaminated substrate and the solution. On the other hand, the samples that were stored in wet solution showed a minimal loss of hydrophilicity, suggesting that the wet storage might promote superficial protection against the salivary pellicle formation by keeping the surface hydrophilicity almost unchanged.

For this reason, the alterations found in this study caused by the early interaction with saliva revealed important findings that may compromise the beneficial effects provided by hydrophilic-nanotextured surfaces when they are not stored in wet environments. Following the present results, not only the specific additional surface treatment reported here (reactive plasma) can be compromised after saliva interaction, but also several hydrophilic treatments reported by other investigations applying UV-lights, heating systems or different plasma methodologies might be prejudiced when exposed to the same conditions [15,55,56]. Based on the present results and corroborating previous studies [10,21,22,23,24,25], proper methods for storage and packing of implants presenting hydrophilic surfaces might be determinant to maintain their surface properties until their application.

### 3.3. Cellular Viability

In order to investigate basic biological responses, a murine pre-osteoblast cell line (MC3T3-E1) was used to perform a MTT assay at 24 h and 48 h. Following the chemical–physical surface alterations verified before the biological assay, possible differences in osteoblastic response on the anodized hydrophilic surfaces exposed to saliva were investigated.

**Figure 5:**
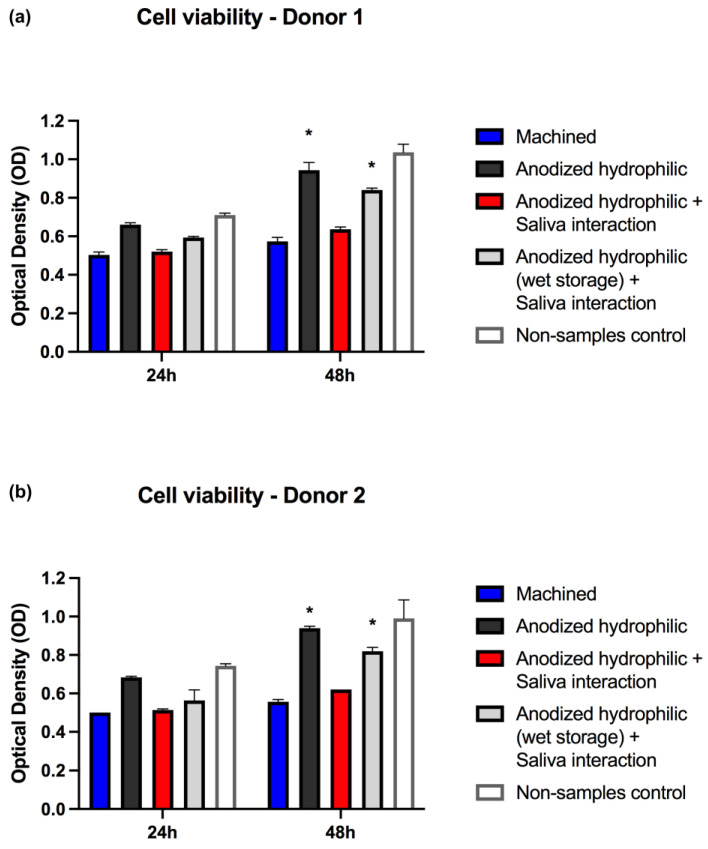
Cell viabilities analysis for the different groups of surface treatments and storage protocols, including two different analyses with two saliva samples (**a**,**b**). * Asterisks signaling significance (*p* < 0.05) when compared to machined surfaces and anodized hydrophilic + saliva interaction surfaces at 48 h.

The cell viability results showed similarities between the two different saliva samples tested for surface contamination. The anodized hydrophilic surface with saliva interaction and without wet storage presented impaired cell viability at 48 h (*p* < 0.05); in addition, the 24 h viability results of saliva-contaminated samples were slightly lower compared to non-contaminated samples (Figure 5). Moreover, the anodized hydrophilic surfaces without saliva contamination demonstrated higher cell viability against all the other groups at 24 h and 48 h; however, statistical significance was demonstrated only at 48 h against the control group and the test group without wet storage. These findings indicate better results of the hydrophilic anodized surface compared to other surfaces (machined) and the importance of direct interaction between substrate-cells without any contamination layer.

Previously reported investigations corroborate that saliva interaction with implant surfaces may compromise the behavior of cells [15,57,58]. The saliva interaction promotes the formation of a thin layer over the surface (salivary pellicle) that changes chemical–physical properties and by consequence alters the interaction between the cells and substrate [16]. Hirota et al. revealed that saliva-contaminated surfaces negatively affected the cell morphology evolution and cell spread capacity in osteoblastic cell cultures, when compared to saliva non-contaminated surfaces [15]. Furthermore, in vivo studies showed that the saliva contamination of dental implants placed in sheep significantly compromised the parameters of osseointegration [48]. Meanwhile, our results revealed a meaningful finding provided by the wet storage before saliva interaction, which might have promoted protection against the direct interaction of saliva contaminants to the substrate, maintaining the surface hydrophilicity in good conditions even after 10 min of contact with saliva.

A single biological assay (cellular viability) was performed in the present investigation, and it may not provide conclusive information about how saliva could impair cell proliferation and evolution. Analysis with simultaneous multiple biological tests and in vivo assays are necessary to develop consistent conclusions about the actual cell behavior after early interaction with saliva-contaminated surfaces. Additionally, saliva collected from donors with different oral conditions or different oral healthcare protocols should be compared in order to understand possible differences in saliva composition. The results demonstrated here clearly showed similarities due to the donor saliva’s similar characteristics. However, it is clear that the physical–chemical alterations showed in the wettability tests here had a direct influence in cell viability and should be further investigated. Finally, the results found on wettability changes and reduced cell viability suggest the need for a careful clinical handling of implants with hydrophilic characteristics in the oral environment, in order not to contaminate surfaces with high reactivity and hydrophilicity with saliva and induce important modifications in surface properties designed to promote faster bone healing.

## 4. Conclusions

Despite the limitations of this pilot in vitro study regarding the application of only one single biological assay and one specific surface treatment, this investigation described a promising methodology for the development of nanotubular surfaces with hydrophilic characteristics for future translation in clinical implant surfaces.

After early saliva interaction, the nanotextured surface did not present significant morphological alterations compared to non-contaminated surfaces. However, statistical changes were exhibited regarding hydrophilic characteristics and cellular viability. The hydrophilic condition of the surfaces stored in common oxygen was negatively influenced by saliva interaction, generating a non-hydrophilic surface; consequently, the cellular viability in pre-osteoblasts cell cultures of contaminated Ti surfaces without wet storage was statistically affected at 48 h. Furthermore, the storage of anodized hydrophilic surfaces in wet conditions indicated a possible surface protection effect against saliva impurities contamination, as all characteristics present in non-contaminated anodized hydrophilic surfaces were identified with close similarities.

Lastly, the present findings suggest that saliva interaction on hydrophilic nanotubular surfaces may induce a substantial loss of hydrophilicity once these surfaces are not stored in wet conditions, suggesting the need to prevent saliva interaction when inserting dental implants with hydrophilic properties in the oral environment. Furthermore, the wet storage of hydrophilic nanotextured surfaces seems to be significant to minimize the chances of impairment of their hydrophilic properties. On the other hand, other biological assays and in vivo experiments should be performed in order to confirm the findings reported in the present investigation.

## Figures and Tables

**Table 1:** Surface groups and their storage protocols before saliva interaction.

Surface Groups	Surface Treatment	Storage Protocol before Saliva Interaction
Machined (control)	Cleaned and polished.	Common air (room temperature).
Anodized hydrophilic	Cleaning, polished, acid-etched, anodized, reactive plasma.	Common air (room temperature).
Anodized hydrophilic + Wet storage	Cleaning, polished, acid-etched, anodized, reactive plasma and wet storage.	Immersed in deionized water and sealed in cell-culture plates (room temperature).
Anodized hydrophilic (interacted with saliva samples 1 or 2)	Cleaning, polished, acid-etched, anodized, reactive plasma.	Common air (room temperature).
Anodized hydrophilic + Wet storage (interacted with saliva samples 1 or 2)	Cleaning, polished, acid-etched, anodized, reactive plasma and wet storage.	Immersed in deionized water and sealed in cell-culture plates (room temperature).

**Table 2:** Roughness parameters before/after saliva interaction on the anodized surfaces.

Surfaces	Roughness Parameters		
	Ra (SD)	Sa (SD)	S_dr_ (SD)
Machined (control)	0.17 ± 0.01 μm	0.19 ± 0.01 μm	1.2 ± 0.1 μm
Anodized hydrophilic	1.25 ± 0.21 μm *	1.37 ± 0.23 μm *	1.74 ± 0.2 μm *
Anodized hydrophilic + saliva interaction (donor 1)	1.11 ± 0.15 μm *	1.21 ± 0.17 μm *	1.54 ± 0.2 μm *
Anodized hydrophilic + saliva interaction (donor 2)	1.09 ± 0.16 μm *	1.19 ± 0.14 μm *	1.53 ± 0.21 μm *
Anodized hydrophilic + wet storage + saliva interaction (donor 1)	1.10 ± 0.25 μm *	1.19 ± 0.24 μm *	1.58 ± 0.25 μm *
Anodized hydrophilic + wet storage + saliva interaction (donor 2)	1.08 ± 0.30 μm *	1.20 ± 0.20 μm *	1.57 ± 0.27 μm *

Data were demonstrated as mean and standard deviation (SD). * *p* < 0.05 shows significance compared to control surface (machined). Ra: arithmetic mean of the initial values of the roughness profile (from the mean line and defined for a profile); Sa: arithmetic mean of the initial values of the roughness area (from the mean plane) (2-D Ra); Sdr: developed surface area ratio (3-D measurement).

**Table 3:** Chemical elements identified by EDS before/after saliva interaction on anodized surfaces.

Chemical Elements (%)	Different Groups					
	Machined (control)	Anodized Hydrophilic	Anodized Hydrophilic + Saliva Interaction (Donor 1)	Anodized Hydrophilic + Saliva Interaction (Donor 2)	Anodized Hydrophilic + Wet Storage + Saliva Interaction (Donor 1)	Anodized Hydrophilic + Wet Storage + Saliva Interaction (Donor 2)
Ti (Titanium)	67.3	52.2	52.8	59.3	59.4	60.5
C (Carbon)	25.2	7.8	35.2	30.7	25	23
O (Oxygen)	7.5	40.0	10	7.8	15	16
Na (Sodium)	-	-	0.6	0.45	0.2	0.2
K (Potassium)	-	-	0.2	0.35	-	-
Ca (Calcium)	-	-	0.7	0.8	0.2	0.15
P (Phosphorus)	-	-	0.5	0.6	0.2	0.15

Obs: “-” means not identified.

## Data Availability

The processed data required to reproduce these findings are available by request to the authors of this study if necessary.
